# Manufacturing Technology of Ceramic Pebbles for Breeding Blanket

**DOI:** 10.3390/ma11050718

**Published:** 2018-05-02

**Authors:** Rosa Lo Frano, Monica Puccini, Eleonora Stefanelli, Daniele Del Serra, Stefano Malquori

**Affiliations:** 1 Department of Civil and Industrial Engineering, University of Pisa, Largo Lucio Lazzarino 2, 56126 Pisa, Italy; eleonora.stefanelli@ing.unipi.it (E.S.); daniele.delserra@ing.unipi.it (D.D.S.); 2 Industrie Bitossi S.p.A, via Pietramarina 53, 50059 Vinci (FI), Italy; malquoris@colorobbia.it

**Keywords:** breeding blanket, fusion reactor, lithium orthosilicate, manufacturing

## Abstract

An open issue for the fusion power reactor is the choice of breeding blanket material. The possible use of Helium-Cooled Pebble Breeder ceramic material in the form of pebble beds is of great interest worldwide as demonstrated by the numerous studies and research on this subject. Lithium orthosilicate (Li_4_SiO_4_) is a promising breeding material investigated in this present study because the neutron capture of Li-6 allows the production of tritium, 6Li (n, t) 4He. Furthermore, lithium orthosilicate has the advantages of low activation characteristics, low thermal expansion coefficient, high thermal conductivity, high density and stability. Even if they are far from the industrial standard, a variety of industrial processes have been proposed for making orthosilicate pebbles with diameters of 0.1–1 mm. However, some manufacturing problems have been observed, such as in the chemical stability (agglomeration phenomena). The aim of this study is to provide a new methodology for the production of pebbles based on the drip casting method, which was jointly developed by the DICI-University of Pisa and Industrie Bitossi. Using this new (and alternative) manufacturing technology, in the field of fusion reactors, appropriately sized ceramic pebbles could be produced for use as tritium breeders.

## 1. Introduction

**Figure 1:**
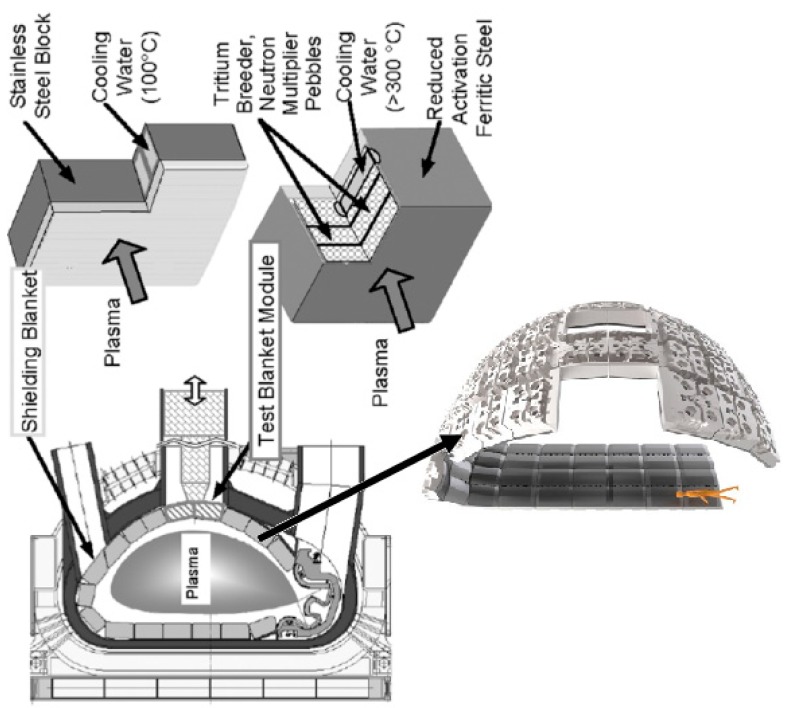
Blanket modules covering the inner walls of the vacuum vessel with zooming of the module structure.

The blanket (Figure 1) is one of the most critical and technically challenging components in a fusion reactor, because together with the divertor, it directly interacts with the hot plasma and has to support a high heat load and an intense neutron flux. Materials have a significant impact on the sizing of the blanket, which influences the power conversion efficiency and the system lifetime. Consequently, the selection of the breeding blanket material is of meaningful importance for the feasibility of fusion technology.

Thus, the major technical efforts in this framework are focused on tritium breeders, which are lithium-containing materials. To identify the most suitable candidate material, a variety of breeding blanket concepts have been considered, such as lithium ceramic breeders, liquid breeders, etc. [1,2,3,4].

In particular, the use of lithium ceramic material in the form of pebble beds attracted considerable interest worldwide, as demonstrated by the numerous studies and research investigating the feasibility of stable fabrication methods and the characterization of pebbles (e.g., stability, thermal conductivity, thermo-mechanical properties, etc.) [5,6,7,8,9,10,11,12,13]. A design solution with lithium ceramic incorporated into fusion blankets, as packed beds of spheres or pebbles, offers several advantages, including simpler assembly into complex geometries, uniform porosity and low temperature sensitivity to cracking or irradiation damage. However, it is evident that the conditions of the breeder blanket in which the pebbles have to operate in are challenging.

In this study, the attention is focused on lithium orthosilicate (Li_4_SiO_4_). This is of interest because lithium has a high density, low thermal expansion coefficient, high stability and high thermal conductivity [5,6]. In addition, a new fabrication method for producing stable and well-sized pebbles of Li_4_SiO_4_ based on the drip casting is presented and discussed.

In the following sections, we will describe the methodological approach and the actual fabrication process. This method is based on the dripping of a slurry at room temperature and this is innovative in the framework of available fabrication processes, which generally require high temperatures. The properly designed experimental device for dripping pebbles is also briefly described.

## 2. Materials and Methods

A variety of industrial processes, even if far from the industrial standard (there is no proof currently), have been proposed for making orthosilicate pebbles with diameters of 0.1–1 mm. The diameter of 0.1–1 mm is an acceptable production characteristic for pebbles to fill the breeder volume of 30–100 m^3^ with a mean packing factor of 60%.

Del Serra et al. [14] analyzed the fabrication processes, the main characteristics of which are summarized in Table 1. They highlighted how the chemical stability, the presence of impurities, the incomplete process phases, etc. are important concerns for obtaining pebble materials with the desired properties. They also analyzed the degradation of pebbles in relation to the manufacturing process, remarking that the hygroscopic nature of lithium ceramic is a critical factor.

As for the melt-spraying process, this process was demonstrated to be unsatisfactory due to the formation on the pebble surface of a second phase (Li_2_SiO_3_) and dendrites [14,15,16,17], which were related to the accumulation of solute and heat ahead of the interface.

The wet method reported by Gao et al. [18], who used Li_2_CO_3_ and SiO_2_, may also be considered to not be satisfactory due to the problems of ensuring the size and roundness of the pebbles. Other fabrication processes, e.g., based on sol-gel technique [19,20], produced high porous (and thus low resistant) pebbles or pebbles with low lithium content (depending on the precursors selected).

### 2.1. Drip Casting

In this study, the forming method used to produce Li_4_SiO_4_ ceramic pebbles is based on drip casting, which is a new process in the field of nuclear fusion material. Therefore, drip casting is a process for producing pebbles by dripping a ceramic suspension through a nozzle plate to form droplets, before hardening the droplets in a saline solution. Moreover, through the modification of the suspension (microstructure) characteristics and the ceramic production process parameters, it is possible to directly modify the properties of pebbles, including mechanical properties, such as compressive resistance, fracture toughness, hardness and abrasion resistance. This process allows the production of stable and well-sized ceramic pebbles, which at the same time also guarantees the sphericity and surface smoothness, the theoretical density and the other properties, such as the resistance, the hardness and fracture toughness, etc., which are traditionally desirable.

#### 2.1.1. Li_4_SiO_4_ Pebbles Fabrication Process

**Figure 2:**
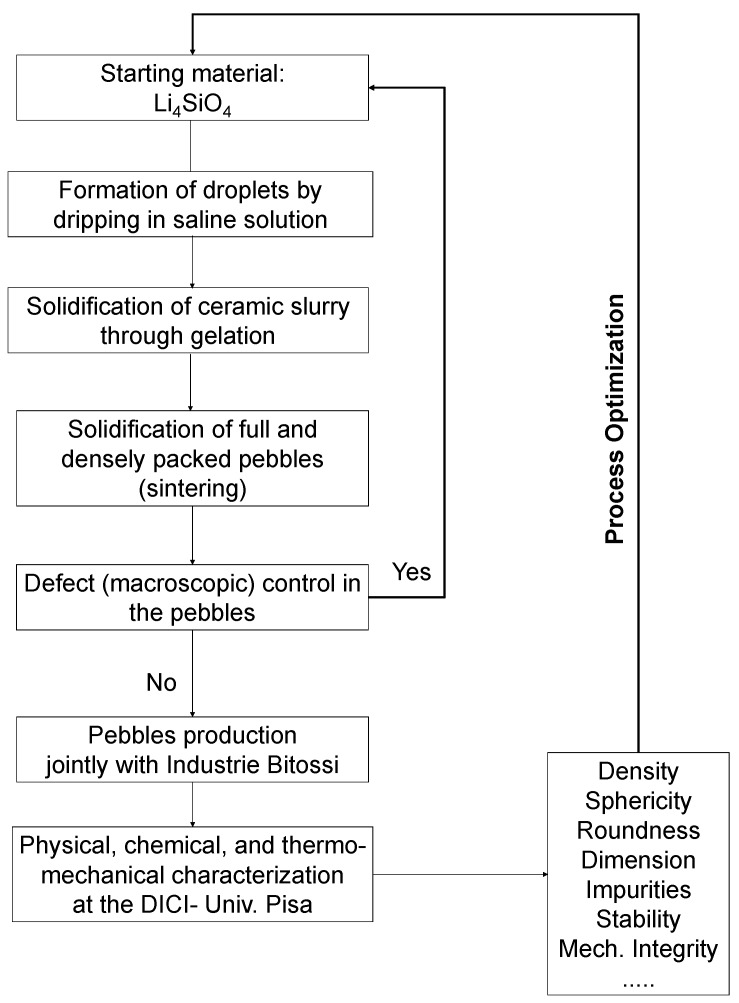
Flow diagram with the main phases of the proposed fabrication process [15].

The innovative character of the new methodology under development at the Department of Civil and Industrial Engineering (DICI) of the University of Pisa jointly with Industrie Bitossi is based on the drip casting method (main phases synthetized in Figure 2), which operates at room temperature.

**Figure 3:**
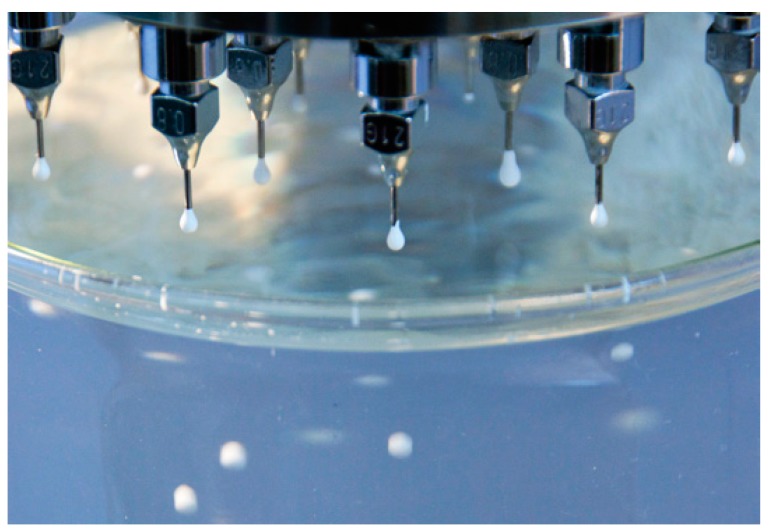
Drip casting process: cross-linking involves the cooperative bonding of divalent metal ions.

Li_4_SiO_4_ in powder form purchased from Albemarle (Frankfurt, Germany) was used as the starting material. First, the process involved the preparation of a Li_4_SiO_4_ aqueous suspension with sodium alginate ((C_6_H_7_NaO_6_)n purchased from Sigma-Aldrich, Darmstadt, Germany). After this, the suspension was forced to pass through a nozzle plate to form droplets. Finally, the droplets were dropped into an aqueous solution of calcium chloride (CaCl_2_ purchased from Sigma-Aldrich, Darmstadt, Germany) where they coagulated in solid spheres (Figure 3). After these steps, the obtained spheres were dried and sintered to form Li_4_SiO_4_ pebbles.

#### 2.1.2. Slurry and CaCl_2_ Solution Preparation

For the slurry preparation, a sodium alginate solution was added to the Li_4_SiO_4_ aqueous suspension. First, 20 wt % of lithium orthosilicate was grinded and dispersed in deionized water for 2 h using a rapid mill equipped with a porcelain alumina jar and alumina balls with 5-mm diameter. This step is necessary to reduce the particle dimensions of Li_4_SiO_4_ powders to a size (medium value of the particle size distribution 500 nm) that ensures a colloidal suspension without the precipitation phenomena, which allows us to obtain a homogeneous dispersion of particles. Second, 5 wt % of sodium alginate was dissolved in deionized water at 80 °C for 2 h using a magnetic stirrer. This temperature helps the dissolution of sodium alginate in water, reducing the viscosity of the solution. After this, the alginate solution was added to Li_4_SiO_4_ suspension in order to obtain a slurry with a global concentration of sodium alginate of 3 wt % (total water basis). The slurry was mixed with a magnetic stirrer for 30 min. Finally, 5 wt % calcium chloride solution was prepared by mixing CaCl_2_ in deionized water for 30 min.

### 2.2. Experimental Apparatus

**Figure 4:**
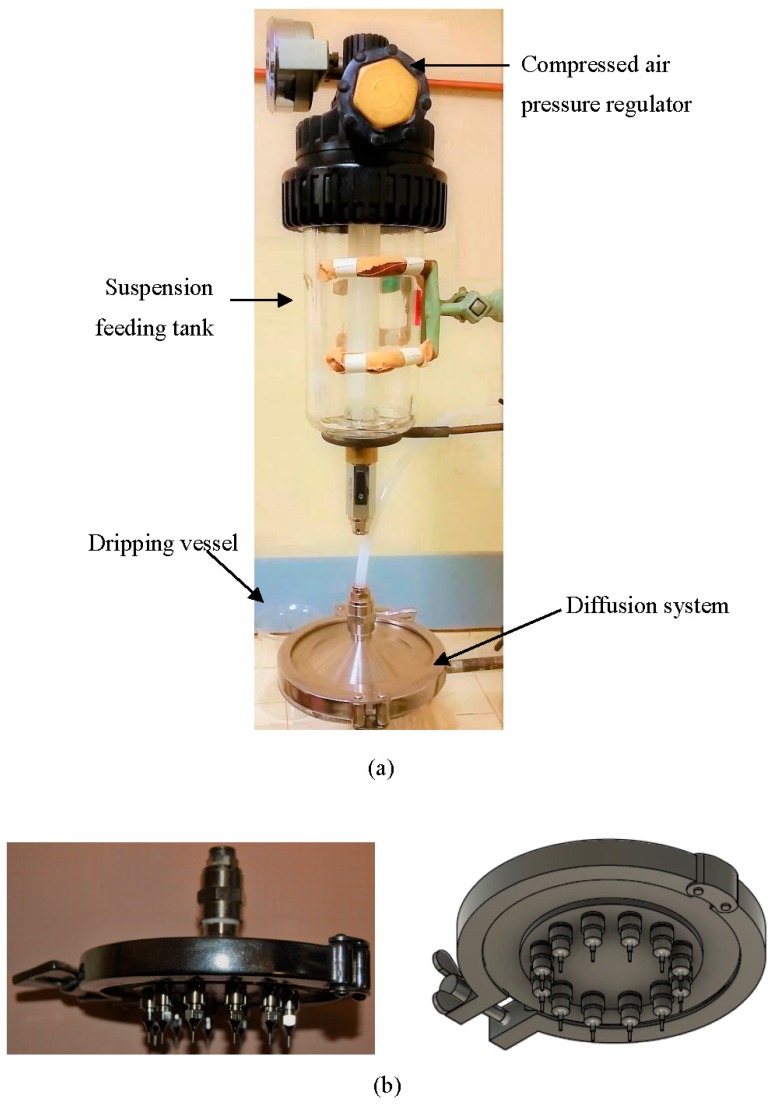
Overview of the experimental device (**a**) with details (photographs and schematic overview) of the dripping sub-systems with the view of the nozzles (**b**).

The experimental test facility shown in Figure 4 has been properly designed and constructed at DICI, University of Pisa. It was composed of four principal elements: a feeding tank containing the Li_4_SiO_4_ suspension, a diffuser disk, a multiple-nozzle plate (nozzles with internal diameter of 0.8 mm) and a collection tank containing the saline solution.

The feeding tank was connected to a compressed air line and equipped with a pressure regulator in order to ensure the dripping of the slurry through the nozzles. A pressure of around 2 atm was used for dripping.

The outlet flow of the Li_4_SiO_4_ suspension was controlled by a manual ball valve. An elevator under the collection tank allowed the regulation of the distance between the nozzles and the reacting solution surface.

Since the device operated at an ambient temperature and at about atmospheric pressure, no particular criteria were required for its design.

The nozzles’ dimension was of a particular size in order to obtain pebbles of 1-mm diameter as optimum after sintering. In fact, the radius of the pebbles is dictated by the requirements for tritium release characteristics and the packing factor of the blanket.

## 3. Experimental

The prepared suspension contained in the suspension vessel was forced towards the ducts, which connected the former to the dripping sub-system by applying a slight pressure.

Therefore, the mixture was conveyed to the diffusion system, the truncated conical shape of which facilitated the transfer to the dripping system. As the slurry ran through the nozzles, droplets formed and fell into the CaCl_2_ solution, forming semi-rigid gelled spheres. This could be due to the polymerization of sodium alginate monomers, which creates a three-dimensional network and leads to the solidification of the ceramic slurry.

After dripping, the obtained spheres were kept in the CaCl_2_ solution for 2 h in order to ensure complete gelation, before being collected with a metallic mesh.

The principle of the drip casting technique involves the solidification of the Li_4_SiO_4_ suspension by the formation of cross-links among the alginate polymeric chains. When the suspension drops into the saline solution, the alginate sodium cations are replaced by divalent cations (Ca^2+^), before immediate irreversible gelation takes place.

**Figure 5:**
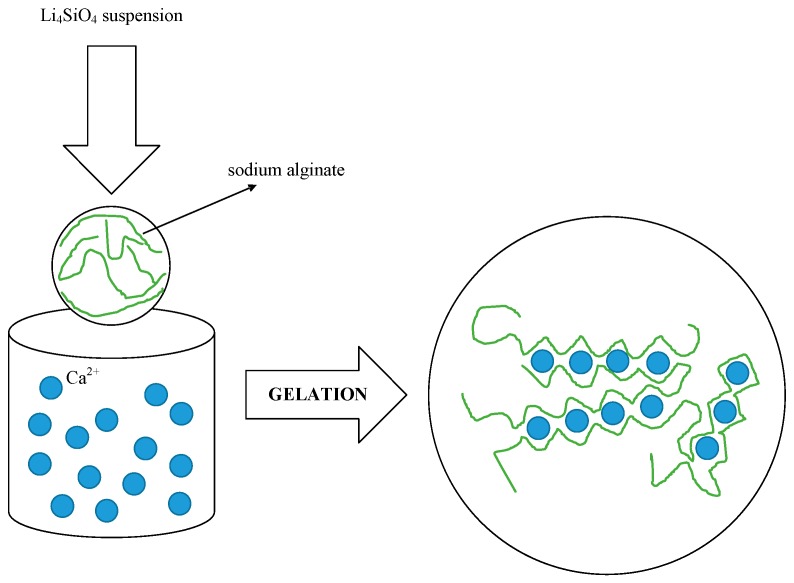
Drip-casting gelation process with sodium alginate.

Li_4_SiO_4_ particles are maintained in a three-dimensional network. The gelation reaction scheme is shown in Equation (1) and represented in Figure 5.

2 Na-alginate + Ca^2+^ → Ca-alginate + 2 Na^+^ (1)

The obtained spheres were dried at 40 °C for 4 h, before being sintered in air at 600 °C for 2 h with a heating rate of 10 °C/min. Finally, they were left to cool at room temperature. During sintering, the calcium alginate network was combusted.

**Figure 6:**
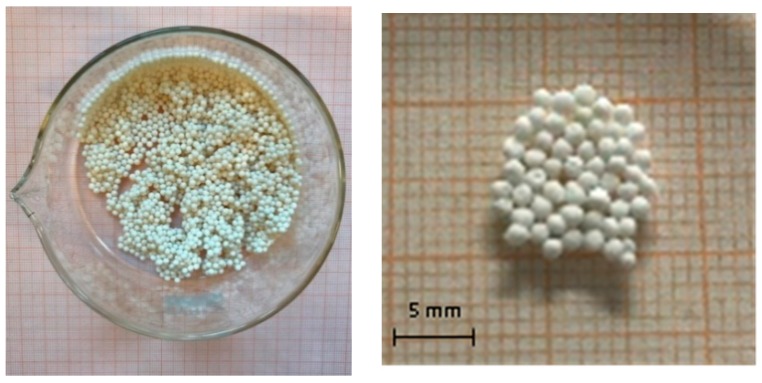
Li_4_SiO_4_ pebbles obtained by the drip casting process at DICI laboratory.

**Figure 7:**
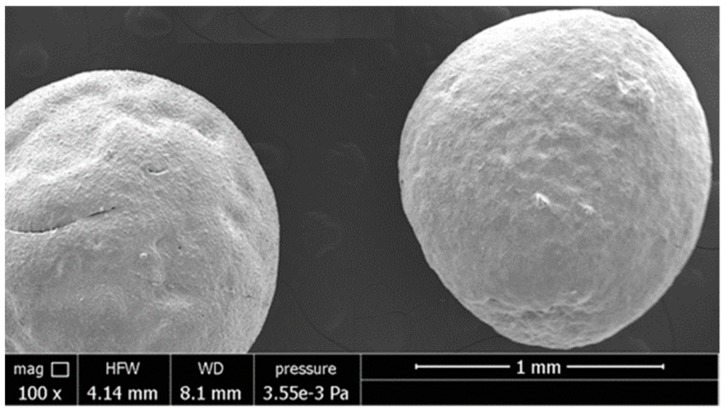
SEM images of Li_4_SiO_4_ pebbles obtained by drip casting process.

The Li_4_SiO_4_ pebbles obtained with this fabrication process seem full and densely packed. As shown in Figure 6 and Figure 7, they have regular roundness with the same sphericity grade and they do not exhibit macroscopic defects. The pebbles produced demonstrate the feasibility of the drip casting process. Nevertheless, the optimization of this process is, in any case, still necessary.

In the future, we plan to perform the thermo-mechanical characterization of the obtained pebbles according to the methodological procedure given in the flow diagram of the previous Figure 2. This characterization will allow us to determine density, microstructure and crystal form, morphology, the mechanical strength (by performing cyclic compression tests and crushing tests with Instron apparatus) and the effective thermal conductivity. The knowledge of this latter, which may be influenced greatly by the deformations, is an essential key issue to be investigated for a proper thermo-mechanical design of the blanket and for assessing heat transfer processes.

## 4. Summary

The improvement of the breeding material is essential for the design and development of the fusion reactor, which requires a high tritium breeding capability of the blanket.

In this study, we proposed the drip casting process as the alternative technology to the high-temperature methods for producing ceramic pebbles to be used in the fusion reactor. This fabrication method allowed us to obtain stable Li_4_SiO_4_ with more accurate and controlled spheroidal particle sizes (and to pave the way towards the industrial standard). The drip casting technology is an efficient manufacturing process since it permits the modification of the initial setting of suspension to suit specific purposes and accordingly, to produce pebbles of different properties. The proposed process allows us to control not only the geometry, but also the density, coherence and chemical stability of the pebbles (responsible for the detriment of pebbles properties).

Ceramic pebbles were produced by dripping a suspension of Li_4_SiO_4_, sodium alginate and water into an aqueous solution of CaCl_2_, which was sintered afterwards. The obtained pebbles were all of a similar size, with a diameter of about 1 mm. They showed regular roundness, without macroscopic defects.

The present work suggests that the drip casting process can be adapted for producing ceramic pebbles. Nevertheless, process optimization and characterization of pebble properties (physical, chemical and thermo-mechanical characterization) are necessary. Both these two activities are planned to be performed in the forthcoming future.

## Figures and Tables

**Table 1:** Pebble fabrication processes [15].

Fabrication Process	Raw Materials	Main Process Phases	Diameter and Density
Melt-spraying [15,16,17,18]	LiOH + SiO_2_	1. Melting	Φ: 0.25 ÷ 0.63 mm (50%) ρ > 0.90 TD
		2. Dropping	
		3. Quenching	
		4. Annealing (2 h at 1000 °C)	
	Li_2_CO_3_ + SiO_2_	1. Melting	Φ: 0.8 ÷ 1.0 mm (50%) ρ > 0.90 TD
		2. Dropping	
		3. Quenching	
		4. Annealing (2 h at 1000 °C)	
Sol-gel [19,20]	LiOH in citric acid suspension (C_6_H_8_O_7_) + SiO_2_ (aerosol)	1. LiOH + citric acid + water	Φ: 1.2 mm ρ = 0.74 TD
		2. Silica addition	
		3. Vaporizing at 70 °C	
		4. Dropping into acetone	
		5. Calcining + sintering (4 h at 900 °C)	
Capillary-based microfluidic [21]	C_2_H_3_O_2_Li·2H_2_O + SiO_2 _lithium acetate dihydrate	1. Stirring raw materials.	Φ: 0.84 mm ρ = 0.82 TD
		2. Inlet T junction with silicon oil	
		3. Vaporization and drying	
		4. Silicon oil removal (72 h at 120 °C)	
		5. Calcining + sintering (4 h at 750 °C)	
Wet process with substitution [15]	LMT powder	1. Dropping LMT + sodium alginate + 4HF in zinc chloride	Φ: 0.238 mm ρ = 0.89 TD
		2. Calcining	
		3. Sintering	
